# Emergency surgery for Meckel's diverticulum

**DOI:** 10.1186/1749-7922-3-27

**Published:** 2008-08-13

**Authors:** Raj Kumar Sharma, Vir Kumar Jain

**Affiliations:** 1 Department of General Surgery, Rajendra Institute of Medical Sciences, Ranchi, India

## Abstract

The current work attempts to highlight the various life threatening complications of Meckel's diverticulum and to present the surgical strategies used in the emergency conditions so far in the form of a review of the works presented in the literature. Our aim behind this presentation is to cover the possible indications, methods, their complications and the outcome of these surgical techniques. For this, we made an extensive literature search using Google and Pubmed with the words-"Meckel's diverticulum", "Complications", "Management" and "Emergency surgery". All the relevant articles containing the surgical aspects of symptomatic Meckel's diverticulum till May 2008 were collected and analyzed. Meckel's diverticulum is the remains of the prenatal yolkstalk (Vitellointestinal duct). Although it generally remains silent but life threatening complications may arise making it an important structure for having a detailed knowledge of its anatomical and pathophysiological properties to deal with such complications.

## Introduction

Meckel's diverticulum was first described by Fabricius Hildanus in 1598. The name derives from the German anatomist Johann Friedrich Meckel who described the embryological and pathological features in 1809 [1]. Although it generally remains silent but life threatening complications may arise making it an important structure for having a detailed knowledge of its anatomical and pathophysiological properties to deal with such complications.

The literature is replete with the description of Meckel's diverticulum for its clinical presentations and complications. A search of Pubmed with the word "Meckel's diverticulum" shows 2835 articles till May 2008. However, the emergency surgery part of the symptomatic Meckel's diverticulum is still deficient in the literature. This seems to be so because most of the articles have been in the form of case reports or case series, so that the management strategies have been highly individualized based on both the patients condition and on the surgeons perspective. This seems to be the appropriate approach in an emergency condition. We intend to underscore some general principles used by these surgeons in their emergency surgeries. The current work attempts to highlight the various life threatening complications of Meckel's diverticulum and to present the surgical strategies used in the emergency conditions so far in the form of a review of the works presented in the literature. Our aim behind this presentation is to cover the possible indications, methods, their complications and the outcome of these surgical techniques. For this, we made an extensive literature search using Google and Pubmed with the words-"Meckel's diverticulum", "Complications", "Management" and "Emergency surgery". All the relevant articles containing the surgical aspects of symptomatic Meckel's diverticulum till May 2008 were collected and analyzed.

**Figure 1:**
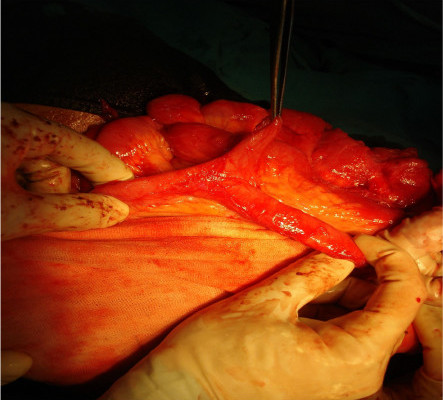
Incidentally found Meckel's diverticulum.

Meckel's diverticulum is the remain of the prenatal yolkstalk (Vitellointestinal duct). The yolk sac of the developing embryo is connected to the primitive gut by the yolk stalk or vitelline (i.e. omphalomesenteric) duct. This structure normally regresses between the fifth and seventh weeks of fetal life. If this process of regression fails, various anomalies can occur. The spectrum of defects includes a Meckel diverticulum, a fibrous cord attaching the distal ileum to the abdominal wall, an umbilical-intestinal fistula, a mucosa-lined cyst, or an umbilical sinus. Of these, Meckel's diverticulum is the most common congenital anomaly of the gastrointestinal tract in humans occurring in approximately 2% of the population with equal incidence in males and females [2]. It is located on the antimesentric border of the ileum 45 to 60 cm proximal to the ileocecal valve and is usually 3–5 cm long [3] [fig 1]. It possesses all the three layers of the intestinal wall and has its own blood supply from the superior mesenteric artery, which makes it vulnerable to infection and obstruction like appendix[3]. Since cell lining of vitelline duct are pluripotent, we may get heterotopic gastric mucosa (50%), pancreatic mucosa(5%) and less commonly colonic mucosa, endometriosis, hepatobiliary tissue, which are responsible for other complications like hemorrhage, chronic peptic ulceration and perforation [2,4,5].

Majority of the meckel's diverticulum remain silent and are diagnosed incidentally during small bowel contrast study, laparoscopy or laparotomy done for unrelated conditions, or until complications arise from the diverticulum [6-8]. A person with Meckel's diverticulum has a 4 – 6% lifetime risk of developing a complication [1,9]. The most common clinical presentation is gastrointestinal bleeding, which occurs in 25% – 50% of the patients having complication [3]. Other complications include obstruction, intussusception, diverticulitis and perforation. Age wise statistics reveals that hemorrhage is the most common presentation in children aged 2 years or younger[3,10] and intestinal obstruction being the commonest among adults[11], although some studies have found reverse [12]. Overall, the complications have been found more common in males, with the ratios varying in different studies from 1.8:1 to 3:1 [13-15]. The pathogenesis of ulceration in a Meckel's diverticulum is secondary to peptic ulceration from heterotopic gastric mucosa. Although colonization of Helicobacter pylori in this ectopic gastric mucosa has been reported but its role in pathogenesis of complication is yet to established [16].

## Complications and their managements

### Bleeding

Lower gastrointestinal hemorrhage is the most common presentation in children with a symptomatic Meckel diverticulum, with incidence rates recorded as high as 50% [17]. The average age of presentation is 2 years but it may occur in older children and adults [3,10]. The presence of heterotopic gastric and pancreatic mucosa within the Meckel's diverticulum, which secretes acid and highly alkaline pancreatic secretion respectively, causing ulceration of adjacent ileal mucosa is the main pathophysiology behind this. However, in adults, other rare causes of bleeding from Meckel's diverticulum have been noted which include the stromal tumors of the same [14]. Children often present with dark red or maroon stools or stools with blood or mucus, whereas adults usually present with melena and crampy abdominal pain. This is perhaps attributable to slower colonic transit time in adults [18]. The bleeding is typically painless and it may be brisk or massive giving the appearance of stool a bright red, brick red or black. Other causes of lower gastrointestinal bleeding in children which include polyps, clotting disorders, arteriovenous malformations, and Crohn's disease, need to be excluded by proper investigations. Technetium -99m pertechnetate radioisotope scintigraphy has been utilized universally for the diagnosis of bleeding Meckel's diverticulum and is at present the investigation of choice in a suspected Meckel diverticulum bleed [19]. In this method the injected radioisotope is readily taken up by the ectopic gastric mucosa within the Meckel diverticulum. The diagnostic sensitivity has been reported as high as 85% with a specificity of 95% and accuracy of 90% in the pediatric age group[2]. The accuracy of such scanning may be increased with the use of pentagastrin (to stimulate the uptake of the radioisotope), histamine-blockers (to inhibit the secretion of the pertechnetate once it is taken up), and glucagon (to inhibit peristalsis and thereby decrease "wash-out" of the pertechnetate). Capsule endoscopy has proved to be of diagnostic value in some cases of bleeding Meckel's diverticulum, however, the reports are very few and a concluding statement regarding its diagnostic value cannot be made at this time[20-22]. Colonoscopy cannot diagnose the bleeding from a Meckel's diverticulum because the colonoscope usually cannot reach the part of the small intestine in which the Meckel's diverticulum is located. But we can think of this pathology when we get blood filled colon without another source of bleeding particularly if accompanied by an abnormal radioisotope scan. Angiography may be useful in the evaluation of an adult patient with occult or intermittent gastrointestinal bleeding for the localization of the site of bleeding, specific diagnosis, and therapeutic preoperative embolization. A vascular blush may also be identified at the site of the Meckel diverticulum. When active hemorrhage is occurring at the time of angiography, luminal extravasation of contrast material will be present [4,23].

When bleeding is massive and can not be controlled by conservative methods, then this is an emergency situation which needs to be dealt promptly with surgical resection of Meckel's diverticulum after initial resuscitation of the patient with blood transfusion. The approach can be through laparotomy, laparoscopy or laparoscopic assisted[24,25]. The aim of the surgery is to resect the Meckel's diverticulum, all ectopic gastric mucosa, and any ulcerated adjacent ileum to prevent recurrent bleeding. During surgery if we find a narrow base without any mass in the lumen, then a wedge resection of the diverticulum with transverse closure of the ileum is the ideal method. We can use linear stapler in this situation to close the ileum. But when the base is wide or mass of ectopic tissue is palpable or when there is inflammation, it is preferable to resect the involved bowel followed by end-to-end ileoileostomy [4]. The rationale of this procedure is derived from the observation that in short Meckel's diverticulum, ectopic mucosa can be seen even in the proximal portion, as compared to long diverticulum where the mucosa has been found mostly in the apical region [26].

### Intestinal obstruction

Intestinal obstruction due to Meckel's diverticulum is the most common presentation in adult and is the second most common in children[11,10]. There are various mechanism by which it can cause intestinal obstruction like (a) Volvulus of small intestine around a fibrous band extending from Meckel's diverticulum to umbilicus. (b) Intussusception – in which Meckel diverticulum sags into the bowel lumen and then serves as a lead point to allow telescoping of the small intestine into first the distal ileum and then in to the large intestine causing ileo-ileal and ileocolic type of intussusception. (c) Littre's hernia – Incarceration of the diverticulum in hernia, (inguinal and femoral) causing intestinal obstruction. (d) Entrapment of small bowel beneath the blood supply of the diverticulum, also known as a mesodiverticular band (e) Stricture secondary to chronic diverticulitis [4] (f) Meckel's diverticulum lithiasis – The formation of stone in Meckel's diverticulum can cause small bowel obstruction by two mechanisms; firstly, it can cause impaction in the terminal ileum after its extrusion from the diverticulum and secondly by promoting local inflammation of the diverticulum and intussusception [27,28] (g) Band extending between the diverticulum and the base of the mesentery, forming a loop in which a part of ileum may get stuck causing obstruction [29]. In our experiences we encountered one such case in which this band forming mechanism was associated with another rare complication of gangrenous change[30]. (h) Other mechanisms involve rare causes of obstruction like tumors (Lipomas, Carcinoid tumors and others), impacted meconium in neonates causing inflammatory adhesions of Meckel's diverticulum to surrounding structures leading to volvulus [31], cecal volvulus around the band extending from Meckel's diverticulum to umbilicus [32], gall stone ileus [33], obstruction secondary to phytobezoar formation in Meckel's diverticulum [22].

Whatever be the cause of obstruction, the presentation is strikingly similar. The patient typically presents with the features of small bowel obstruction like absolute constipation, spasmodic abdominal pain, vomiting which may be bilious and abdominal distention. In case of intussusception we may get the features of acute obstruction associated with an urge to defecate, early vomiting and occasionally, the passage of the classical currant jelly stool[2]. Plain x-ray abdomen may reveal dilated bowel loops and multiple air fluid levels. If this condition is left untreated, it leads to strangulation and ischemic necrosis of the wall of the bowel loop. Gas under diaphragm on plain erect x-ray may be found in that situation. Hence intestinal obstruction should be treated as an emergency situation warranting immediate exploratory laparotomy after initial resuscitation. During exploration if we get volvulus around a fibrous band, untwisting of bowel along with division of band should be done. In case of intussusception, attempts to reduce such mass may be difficult, warranting resection of intussuscepted mass followed by primary anastomosis. However in Litter's hernia, Meckel's diverticulum should be resected after reducing it followed by hernial repair[34]. For a mesodiverticular band, the small bowel is reduced, and the diverticulum along with its blood supply should be resected. Enterolith in Meckel diverticulum should be resected en bloc with primary anastomosis[28]. Thus in cases of intestinal obstruction, the main aim of surgery is still to remove the culprit i.e. Meckel diverticulum along with correction of associated pathology, independent of the chosen surgical approach being either open or laparoscopic.

### Diverticulitis

Diverticulitis represents 20% of the symptomatic Meckel's diverticulum[4] and is common in adult patients [2,3,11]. Clinical manifestation mimics acute appendicitis and should be considered in the differential diagnosis of a patient with right lower quadrant pain. This feature necessitates the exploration of distal ileum when normal looking appendix is found during operation of suspected acute appendicitis. Pathophysiology is analogous to that of acute appendicitis, with inflammation being secondary to stasis and bacterial infection, which occurs due to the obstruction of the lumen by Enterolith or foreign body or by parasites (Ascaris lumbricoides or Taenia saginata) [35]. Alternatively, peptic ulceration of ileal mucosa due to ectopic gastric mucosa can cause diverticulitis. It may also result from diverticular torsion that causes secondary ischemia and inflammatory change [36]. If this condition is left untreated, it usually leads to perforation and peritonitis.

This condition should be dealt with a surgical approach that can be open or laparoscopic, with resection of diverticulum at its base and closure perpendicular to the axis of intestine to minimize the risk of subsequent stenosis. And if perforation has occurred, thorough peritoneal toileting is done after resection.

### Perforation

It is difficult to diagnose the site of perforation prior to exploration although duodenal and ileal perforation can be distinguish to a lesser extent by observing the nature of the aspirates from abdomen i.e. whether it is bilious or feculent. Various etiologies that can lead to perforation of Meckel's diverticulum are (a) Progression of diverticulitis, (b) Ulceration of adjacent ileal mucosa secondary to acid produced by ectopic gastric mucosa, (c) Secondary to ingested foreign body like fish bone [37,38], chicken bone and bay leaf [39], (d) Traumatic [40]. (e) Perforation associated with tumors like Leiomyoma in Meckel's diverticulum has also been reported [41]. It typically presents with the features similar to that of the perforation of other hollow viscera, with features of either localized or generalized peritonitis. Perforation of Meckel's diverticulum is usually managed by initial resuscitation and antibiotics followed by diverticulectomy or segmental resection along with peritoneal irrigation.

### Tumor

Tumors in Meckel's diverticulum are very rare occurrences, with incidence of only 0.5% to 1.9% [42]. These tumors can be benign or malignant. Lipoma, Neuromuscular and vascular hamartoma are among the benign group [43-45]. In the malignant group, carcinoids are the most common tumor occurring with 44% of incidence [46,42]. Others are mesenchymal tumors (including gastro intestinal stromal tumors, leiomyosarcomas and peripheral nerve sheath tumors 35%), adenocarcinomas (16%) [42] and Desmoplastic small round cell tumor [47,46,50].

These tumors can have various manifestations like acute abdominal pain, perforation, bleeding, intussusception and intestinal obstruction making it an emergency situation [45,18]. Lipoma can be dealt with simple diverticulectomy. Since Carcinoid is associated with metastasis early in course (in 25% of cases), solitary, localized, asymptomatic nodules less than 1 cm are generally managed with diverticulectomy or segmental resection. Larger or multiple lesions require wide excision of bowel and mesentery, and hepatic resection may be required for metastatic disease [51,21].

Method of performing open diverticulectomy or segmental resection.

As stated above the treatment of symptomatic Meckel's diverticulum should be prompt surgical resection of the diverticulum or resection of segment of adjacent ileum bearing the diverticulum. Segmental ileal resection is required for the treatment of patients with bleeding because bleeding site is usually in the adjacent ileum.

After opening the abdomen either through midline or right lower incision, cecum and terminal ileum is identified. Ileum is followed proximally where we find Meckel's diverticulum approximately 2 feet from the ileocecal valve. After delivering the diverticulum with ileum into the wound, if mesodiverticulum found, it should be divided and ligated between the clamps. The lumen of diverticulum is emptied of its contents and the base is clamped with two noncrushing clamps and then excised between the clamps. Then the inner layer is stitched in full thickness with 000 vicryl in continuous manner. When this layer is complete the clamp is removed and second seromuscular layer is stitched with inverting interrupted Lembert sutures of 000 mersilk. However, this is an old procedure and trend has changed towards single extra mucosal seromuscular closure. This can be further simplified if stapling device is available [1]. With the advent of endostapling, these procedures are now readily done using the laparoscope.

If the indication of diverticulectomy is bleeding then segmental ileal resection should be done. It is also indicated if tumor is detected or if the base is inflamed or perforated. The diverticulum is excised along with 2 to 3 cm of adjacent ileum. Then single layer end- to- end anastomosis is performed using 000 mersilk and lumen is tested for its patency between thumb and index finger.

Role of laparoscopic surgery in the management of complicated Meckel's diverticulum.

Laparoscopy as a minimally invasive approach has emerged as both diagnostic as well as therapeutic means to deal with various surgical conditions including Meckel's diverticulum. Its ability to visualize whole of the abdomen makes it a diagnostic choice for various undiagnosed intraabdominal pathologies. There are several studies stating the safe and effective use of laparoscopy in case of complicated Meckel's diverticulum [52,25,55]. It can be used in undiagnosed acute abdominal pain, in obstruction [56-58] and perforation [53,58]. But its role in removing bleeding Meckel's diverticulum has been argued by some due to the fact that, the base of the diverticulum and the ileum cannot be palpated, hence ectopic mucosa could be left behind thus leaving the risk of recurrence, but others have successfully dealt with this situation [53,59,60]. The criteria of external appearance to deal with such condition has been studied, with conclusion that long diverticula can be removed by simple transverse resection with a stapling device because long diverticula usually have ectopic tissue at its distal end, but in short diverticula it can occur in almost any area so ileal resection with end-to-end anastomosis or wedge resection after exteriorization is recommended in short type of diverticula [26]. Another study presented the external appearances in terms of height to diameter ratio (HDR) of 2 as a cut off value to decide whether to do simple transverse resection or ileal resection with end-to-end anastomosis [61]. The development of endostapling devices has made the resection safer, faster and more efficient. The most important advantage is its simultaneous cutting and closing properties making the chance of contamination very less [62].

Generally the laparoscopic approach is same as for the appendicectomy. It involves the creation of pneumoperitoneum under direct vision followed by placement of the 10 mm umbilical trocar, 5 mm suprapubic trocar and 5 mm trocar in right lower quadrant of the abdomen based on the principle of "triangulation". Suprapubic port is used for the 5 mm (30°) laparoscope, umbilical port for the right hand instrument and right lower quadrant port for the left hand instrument. If the decision of resection is made then, the 10 mm umbilical port can be enlarged to 12 mm port for the use of endostapler, which is fired at the base of the diverticulum perpendicular to the long axis of the ileum[62]. Alternatively it can also be done by bringing the small bowel out through the enlarged umbilical port site for the palpation and resection.

Some complications of Meckel's diverticulum often need additional laparoscopic interventions. For example, the management of volvulus and intussusception involve techniques like laparoscopic derevolving and desussception respectively. [63,64,42]. Similarly, laparoscopic approaches have greatly simplified the management of Littre's hernia. Both excision of the Meckel's diverticulum using an endoscopic stapling device and repair of this hernia with Permacol, or other meshes have been done, which has been recorded as case reports[34,65,17].

Thus in the present scenario, as compared to the conventional laparotomy, the laparoscopic management of the complicated Meckel's diverticulum has been claimed to be safe, cost effective and efficient, with added advantages of precise operative diagnosis, fewer complications and shorter recovery period [53,58].

## Limitation of laparoscopic surgery

Although it has been substantially proved that laparoscopic surgery is safe and efficient, having all other advantages of minimally invasive surgery, still it has some limitations. The most important one is its unavailability, this being specially true in developing countries where it also gives burden of high cost.

Another technical limitation during surgery is confrontation with either too short or very broad-based Meckel's diverticulum. If its base is too short there is danger of including too much of the ileum during stapling or leaving behind its part when it is of very broad base [62,55]. Such situations can be dealt with resection of Meckel's diverticulum using a Harmonic scalpel and thereafter closing the enterotomy with intracorporeal vicryl sutures. Another safe method to accomplish excision is to exteriorize the diverticulum via a minilaparotomy incision, resection and closing the enterotomy with sutures.

## Laparoscopic assisted surgery

With the advent of laparoscope, both extracorporeal and intracorporeal resection of Meckel's diverticulum may be performed [52]. The indications are almost similar to that of laparoscopic surgery but it scores over it in terms of some additional benefits. In laparoscopic-assisted transumbilical Meckel's diverticulectomy (LATUM), a transumbilical 10 mm trocar is inserted in an open fashion, then using 10 mm operative laparoscope, the terminal ileum is exteriorized through umbilicus with an atraumatic instrument and then diverticulectomy or segmental resection can be performed [60]. Thus this technique also allow palpation of Meckel's diverticulum which aids in ruling out any mass or thickening of base thus providing the more complete assessment for presence of any ectopic gastric mucosa [66]. This technique also prevents the use of costly staplers [25,59] making it more cost effective.

## Complication of surgery

Complications are generally the same as that of other operations like bleeding, infection, intra abdominal abscess formation, wound dehiscence, incisional hernia and post operative adhesive intestinal obstruction. Surprisingly, morbidity (20%) and mortality (3%) of diverticulectomy in the asymptomatic group by any of these procedures have been found to be higher than morbidity (13%) and mortality (0%) in the symptomatic group. This highlights the earlier impression that these complications are the sequels of open abdominal surgery rather than of diverticulectomy [67,12]. These complications have to be dealt in the similar ways as for other surgeries. But special emphasis should be given for the recurrence of bleeding which occurs when some of ectopic gastric mucosa are left behind. To prevent this, ileal resection with end-to-end anastomosis should be done to exclude all of the ectopic gastric mucosa.

## Conclusion

Meckel's diverticulum is the most common congenital anomaly of the gastrointestinal tract but the life time risk of developing complications in this vestigial organ is 4 – 6%. Complications which require emergency treatment includes bleeding, obstruction, diverticulitis and perforation and the appropriate knowledge of various pathophysiologies by which a Meckel's diverticulum can cause complication should be kept in mind for the better management and to prevent recurrences. The diagnosis of symptomatic Meckel's diverticulum needs a high degree of suspicion as the preoperative clinical and investigational diagnosis is difficult to be made with accuracy. Open surgery has long been used as an effective method to deal with complicated Meckel's diverticulum. However, in this age of minimally invasive surgery, laparoscopic management of complicated Meckel's diverticulum is safe, cost effective and efficient as proved by various reports and studies. Although both the methods have their own limitations, still the choice of management depends on patients condition, surgeon's experiences, and availability of the laparoscopic instruments.

## Competing interests

The authors declare that they have no competing interests.

## Authors' contributions

RKS conceptualized the review, drafted the review, analyzed the literature where ever was needed. VKJ conducted extensive literature review, manipulated the draft, contributed to the analysis. All Authors' read and approved the final manuscript.

## Supplementary Material

Additional File 1 Statement of consent Click here for file
