# Supplementary figures and images for: Emergency surgery for Meckel's diverticulum
Source: World J Emerg Surg. 2008 Aug 13;3:27. doi: 10.1186/1749-7922-3-27 (PMC2533303; doi:10.1186/1749-7922-3-27)

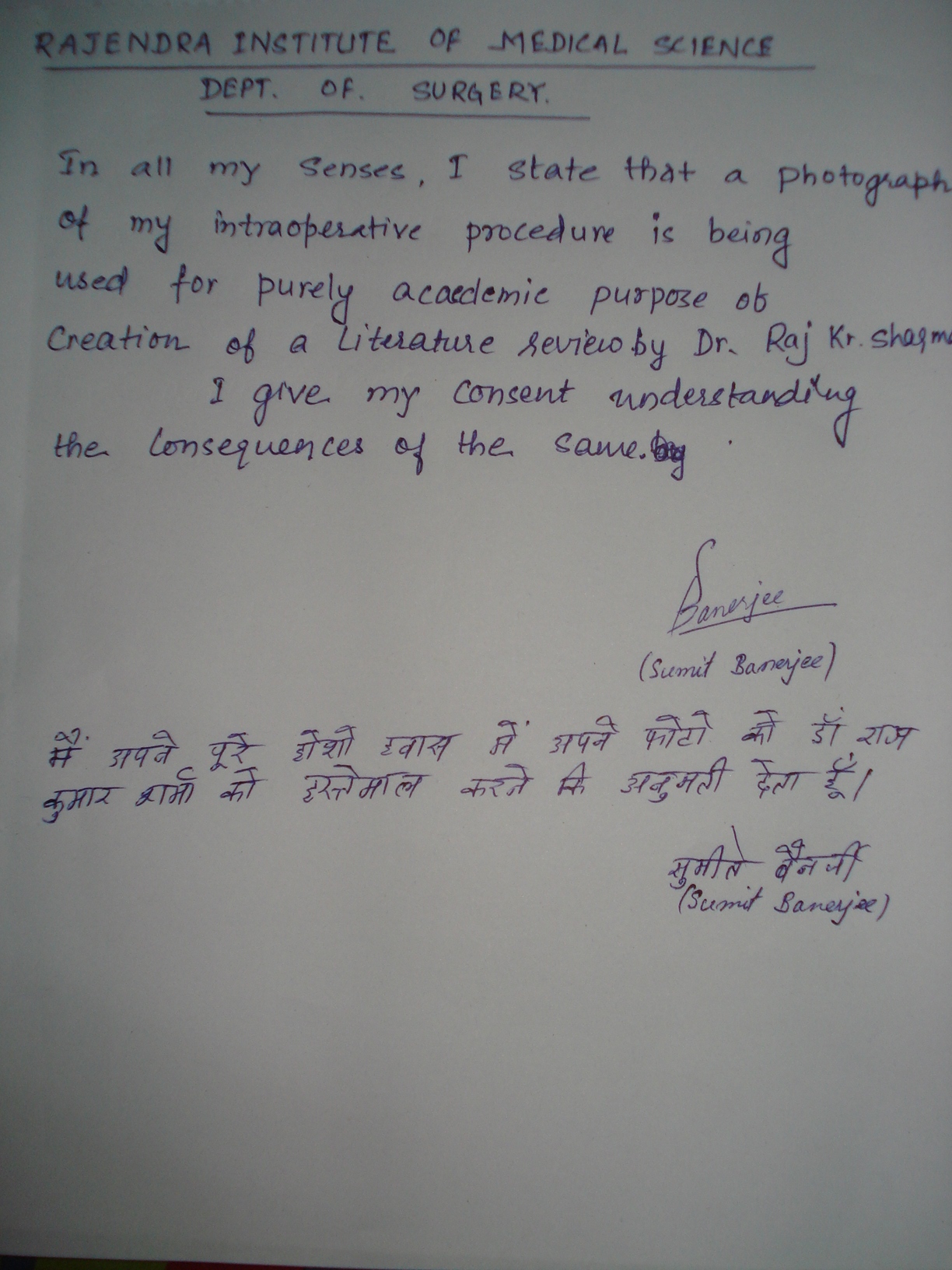

Supplement: Additional File 1 — Statement of consent [file 1749-7922-3-27-S1.jpeg]
